# Accidental Provings of Bellis-Perennis

**Published:** 1895-10

**Authors:** Edmund Carleton

**Affiliations:** New York, N. Y.


### ACCIDENTAL PROVINGS OF BELLIS-PERENNIS.

Edmund Carleton, M. D., New York, N. Y.

The common white daisy has been slightly proved in the rough. Allen's *Encyclopædia* quotes from the *British Journal of Homœopathy* the experiments made by Dr. Thomas, with tincture of the whole plant when in flower; the third dilution; external application of the tincture, and chewing the flowers. Symptoms appeared, of head, stomach, upper extremities, and especially the skin. The latter are as follows:

From Allen's *Encyclopædia of Materia Medica*, Vol. II, page 128.

### Bellis-Perennis. Skin.

"Development of a small boil (after five hours) from external application of tincture. Small boil at the angle of the inferior maxilla, right side (after chewing the flowers). Painful pimple a little behind the angle of left inferior maxilla (third dilution) after third day. Large boil on back of neck, commencing with a dull, aching pain; some difficulty and bruised pain in keeping the head erect; began as a slight pimple, with burning pain in the skin, increasing until, in six days' time, it was very large, of a dark, fiery, purple color, and very sore, burning and aching pain in it, accompanied with headache extending from occiput to sinciput, of a cold, aching character; brain as though contracted in frontal region, dizziness, etc. (after two weeks), from the tincture."

It is my impression that additional provings of *Bellis-perennis* have found their way into our current literature, but have not been collated; and as this scrap has been furnished at very short notice, I have not had time to look them up. One thing is certain. Lately our British colleagues have employed it—shall I say empirically?—constitutionally and locally, for all sorts of traumatisms with great success. It is in high favor in England, and the few American physicians who have employed it are equally enthusiastic over it. Careful provings and clinical demonstrations by Hahnemannians are desirable.

The following fragment was obtained from one of my most intelligent patients, the mother of two youths, a son and daughter: A friend brought a huge bunch of daisies from the country and gave them to the children. They separated the bunch into bouquets, arranged them in vases, added water, and by such means handled the stalks and blossoms. Very soon, probably the next day, numerous little white, itching blisters appeared between the fingers, filled with colorless liquid. The mother being obliged to depend upon her own resources for the time, gave *Aconite* in potency to both the children. This relieved the itching. The daisies were thrown away, being accused of the mischief; and in a few days the hands were well. On the 26th inst. the son came to my office and exhibited his hands. Only the process of desquamation was then visible. No unnatural sensations existed. Close cross-examination by me resulted in confirmation of the history given above. Both he and his sister are well acquainted with *Rhus-toxicodendron* and its action, and are habitually on guard against it. There was no *Rhus* in these cases.

